# Downgrade BI-RADS 4A Patients Using Nomogram Based on Breast Magnetic Resonance Imaging, Ultrasound, and Mammography

**DOI:** 10.3389/fonc.2022.807402

**Published:** 2022-01-27

**Authors:** Yamie Xie, Ying Zhu, Weimin Chai, Shaoyun Zong, Shangyan Xu, Weiwei Zhan, Xiaoxiao Zhang

**Affiliations:** ^1^ Department of Ultrasound, Ruijin Hospital, Shanghai Jiao Tong University School of Medicine, Shanghai, China; ^2^ College of Medicine, Kunming University of Science and Technology, Department of Ultrasound, The First People’s Hospital of Yunnan Province, Kunming, China; ^3^ Department of Radiology, Ruijin Hospital, Shanghai Jiao Tong University School of Medicine, Shanghai, China

**Keywords:** Breast Imaging Reporting and Data System, magnetic resonance imaging, ultrasound, breast tumor, mammography

## Abstract

**Objectives:**

To downgrade BI-RADS 4A patients by constructing a nomogram using R software.

**Materials and Methods:**

A total of 1,717 patients were retrospectively analyzed who underwent preoperative ultrasound, mammography, and magnetic resonance examinations in our hospital from August 2019 to September 2020, and a total of 458 patients of category BI-RADS 4A (mean age, 47 years; range 18–84 years; all women) were included. Multivariable logistic regression was used to screen out the independent influencing parameters that affect the benign and malignant tumors, and the nomogram was constructed by R language to downgrade BI-RADS 4A patients to eligible category.

**Results:**

Of 458 BI-RADS 4A patients, 273 (59.6%) were degraded to category 3. The malignancy rate of these 273 lesions is 1.5% (4/273) (<2%), and the sensitivity reduced to 99.6%, the specificity increased from 4.41% to 45.3%, and the accuracy increased from 63.4% to 78.8%.

**Conclusion:**

By constructing a nomogram, some patients can be downgraded to avoid unnecessary biopsy.

## Introduction

According to statistics, the number of new cases of breast cancer among Chinese women reached 0.42 million in 2020, accounting for 18% of the global breast cancer rate. It ranks first in the incidence of female cancer, and the mortality rate ranks fourth in China (1). The incidence of breast cancer in women has been increasing year by year; female breast cancer has surpassed lung cancer as the most commonly diagnosed cancer, with an estimated 2.3 million new cases (2). Considering the high sensitivity of MRI in the detection of breast diseases, more and more patients will add MRI examination when suspicious lesions are found in mammography or ultrasonography. According to the breast imaging report and data system (BI-RADS), category 4 (2% ≤ malignant rate < 95%); category 5 (malignant rate ≥ 95%) (3, 4). The guidelines recommend that the lesions above or equal to category 4 undergo core needle biopsy to clarify the histopathological type, and the positive predictive value (PPV) spans a large range. An analysis of data from 1.6 million women’s breast cancer surveillance associations showed that 66.8% of biopsy results were benign (5), the positive predictive value (PPV) of 4A patients is less than 10%, a large part of the pathology of biopsy specimens is confirmed to be benign, and the high sensitivity and low specificity lead to unnecessary invasive examinations. Therefore, a better way to stratify and manage patients belonging to category 4 is needed.

The main purpose of this study is to downgrade category 4A lesions to avoid unnecessary biopsy. Since BI-RADS 4B, 4C, and 5 patients are still within the puncture range even if they are degraded, the degrading factors of these patients are not considered in our study.

## Materials and Methods

### Study Participants

This retrospective study was approved by the Ethics Committee of the Ruijin Hospital, Shanghai Jiao Tong University School of Medicine. The consent to participate in the study for patients was waived due to the retrospective study and all identity data of patients are undistinguishable. The study was carried out in conformity to the Declaration of Helsinki (as revised in 2013). From August 2019 to September 2020, 6,312 patients underwent breast surgery or core needle biopsy and obtained clear pathological results, including 2,252 females who went through ultrasound, mammography, and magnetic resonance imaging examinations simultaneously. Among the 2,252 patients, 535 were excluded due to the following reasons: (a) the location of the lesions shown by the three imaging ways was inconsistent (*n* = 35), (b) incomplete clinical data (*n* = 26), (c) part of the images was unclear (*n* = 35), (d) the interval between the three imaging examinations was more than 1 month (*n* = 40), (e) patients who have been diagnosed with BI-RADS category 6 (*n* = 49), and (f) non-mass enhancement (*n* = 350). In the end, 1,717 patients constituted the study group. Among them, there are 458 patients in category 4A, (mean age, 47 years; range 18–88 years; all women) patient characteristics is shown in **Table 1**. Basic BI-RADS classification information of patients is shown in **Table 2**.

**Table 1:** Patient characteristics (all patients are women).

Characteristics	Datum
Age (years)	
Mean ± standard deviation	47.0 ± 12.1
Median*	47 (18–88)
Mass mobility	
Well	360 (78.6)
Poor	98 (21.4)
Hormones	
Use	50 (10.9)
Unused	408 (89.1)
Family history	
Yes	33 (7.2)
No	425 (92.8)
History of breast surgery	
Yes	51 (11.1)
No	407 (88.9)
Tenderness	
Yes	52 (11.4)
No	406 (88.6)
Mass texture	
Soft	148 (32.3)
Hard	310 (67.7)

Unless otherwise indicated, data are numbers of patients, with percentages in parentheses. *Data are the median, with the range in parentheses.

**Table 2:** Basic BI-RADS classification information of patients.

		Benign (%)	Malignant (%)	Total
**BI-RADS**	3	29 (100.0)	0 (0.0)	29
	4A	414 (90.4)	44 (9.6)	458
	4B	110 (57.6)	81 (42.4)	191
	4C	91 (16.8)	450 (88.2)	541
	5	14 (2.8)	484 (97.2)	498
	Total	658 (100.0)	1,059 (100.0)	1,717

Unless otherwise indicated, the value is the number of patients and the percentage in parentheses. A total of 1,717 patients with BI-RADS classification were included, and 458 patients with 4A classification were studied. BI-RADS, Breast Imaging Reporting and Data System.

### Imaging Technique

The mammography examination uses the GE Senographe 2000D machine: the projection positions are mainly in the internal and external oblique position (MLO position) and the head and tail position (CC position). If necessary, local compression magnified irradiation and special body position irradiation were given.

All the breast MRI examinations were performed with a 1.5-T unit (MAGNETOM Aera, Siemens Healthcare) with a dedicated 18-channel phased-array breast coil. The patient was in the prone position, and the breasts were naturally suspended in the breast coil. The scanning range included bilateral axillary and bilateral upper and lower boundaries of the breast. The protocol included axial T1-weighted, T2-weighted fat-suppressed, diffusion-weighted imaging (DWI, b value is 1000 s/mm^2^), T1-weighted fat-suppressed dynamic enhancement scan: 1 stage no enhancement 90 s + 5 stage enhancement (90 s × 5) after injection of 20 ml of gadolinium meglumine, and then the images were uploaded to the PACS system. Postprocessing included T1-weighted subtraction, T1-weighted maximum intensity projection, and subtracted sagittal reconstruction; the apparent diffusion coefficient (ADC) was measured; and the time–signal intensity curve (TIC) was obtained.

The ultrasound system used Mindray Resona 7, a linear array probe, and the frequency is 10.0 to 14.0 MHz. Choose the breast model, the patient takes the supine position, the arm is raised or abducted, and the breast and axilla are fully exposed. Ultrasound examination of the entire breast should be from the posterior axillary line to the parasternal line, with the nipple as the center to scan the entire breast, the nipple-areola complex area, and its affiliated lymph nodes. When checking the blood flow in the lesion, the probe should be placed lightly and not pressurized, to avoid the loss of small blood vessel compression. Shear wave elastography (SWE) is converted to the SWE model when the longest axis view of the lesion is displayed on the 2D image, and the probe should be handled gently.

### Imaging Evaluation

The analyzed images were downloaded from the hospital’s PACS system in DICOM format. Two people engaged in breast research (YZ and YX, with 4 and 12 years of experience in breast diagnosis) are also proficient in ultrasound, mammography, and magnetic resonance diagnostic images. Radiologists, without knowing the pathological results, according to the fifth edition of the ACR BI-RADS lexicon, used the ultrasound images (add elasticity, blood flow, and the maximum diameter of the mass), magnetic resonance images (add ADC, DWI, and subtraction), and mammography images for evaluation. When one of the three images shows an obvious mass, it is defined as masses; otherwise, it is classified as no mass enhancement.

### Statistical Analysis

Continuous variables are expressed as the mean ± standard deviation, and categorical variables are presented as frequencies and percentages. Univariable analyses are performed by Student’s *t*-test or one-way ANOVA when normally distributed, or the Mann–Whitney *U* test when not normally distributed.

For this study, based on the BI-RADS lexicon, the clinical indicators included the patient’s age, history of hormone therapy, the activity of the mass, the mass texture, family history of breast cancer, history of breast surgery, and whether there is tenderness. Variables showing *p* < 0.05 in univariable analysis were considered possible predictors and were entered in multivariable logistic regression. The independent influencing factors of benign and malignant tumors were screened out using multivariable logistic regression.

Convert continuous variables into categorical variables to facilitate the drawing of the nomogram: the best cutoff value is obtained by Youden index (ADC value is 1.035 × 10^-3^ mm^2^/s, SWEmax is 72.61 KPa). Patients were divided into three groups based on their age according to the United States Cancer Screening Guidelines (6) and the epidemiological characteristics of breast cancer in China. The total score of each patient is obtained by assigning each index of the patient and adding up all the scores. The pathological results were used as the “gold standard”, and the area under the receiver operating characteristic curve (AUC) was calculated after determining a cutoff value of total points by analyzing the nomogram. Sensitivity, specificity, and accuracy are calculated: BI-RADS score of 2–3, benign; BI-RADS score 4 or above, malignant. Using the area under the receiver operating characteristic curve (ROC) and the calibration curve, evaluate the diagnostic accuracy. The software SPSS Statistics (version 26.0, USA) and R software (version 4.0.5) were used for data analysis. A *p*-value of <0.05 was considered significantly different.

## Results

### Pathological Features

Of 458 patients, 44 are malignant, namely, 19 cases (43.2%) of invasive ductal carcinoma, 13 cases (29.5%) of ductal carcinoma in situ, 4 cases (9.1%) of papillary carcinoma, 1 case (2.3%) of malignant phyllodes tumor, 1 case (2.3%) of small B-cell lymphoma, 3 cases (6.8%) of invasive lobular carcinoma, 2 cases (4.5%) of lobular carcinoma *in situ*, and 1 case (2.3%) of mucinous carcinoma. In addition, adenopathy, papilloma, fibroadenoma, benign phyllodes tumor, sclerosing adenopathy, and accompanied by ductal dilatation were the most common benign lesions.

### Imaging and Clinical Factors

**Figure 1:**
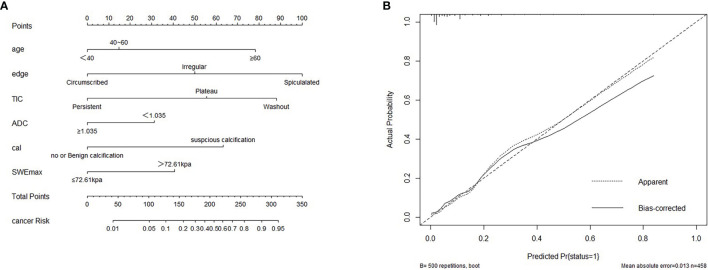
**(A)** Nomogram for predicting benign and malignant mass in category 4A patients. **(B)** Calibration curve based on model. cal, calcification morphology; TIC, time–signal intensity curve; ADC, apparent diffusion coefficient.

**Figure 2:**
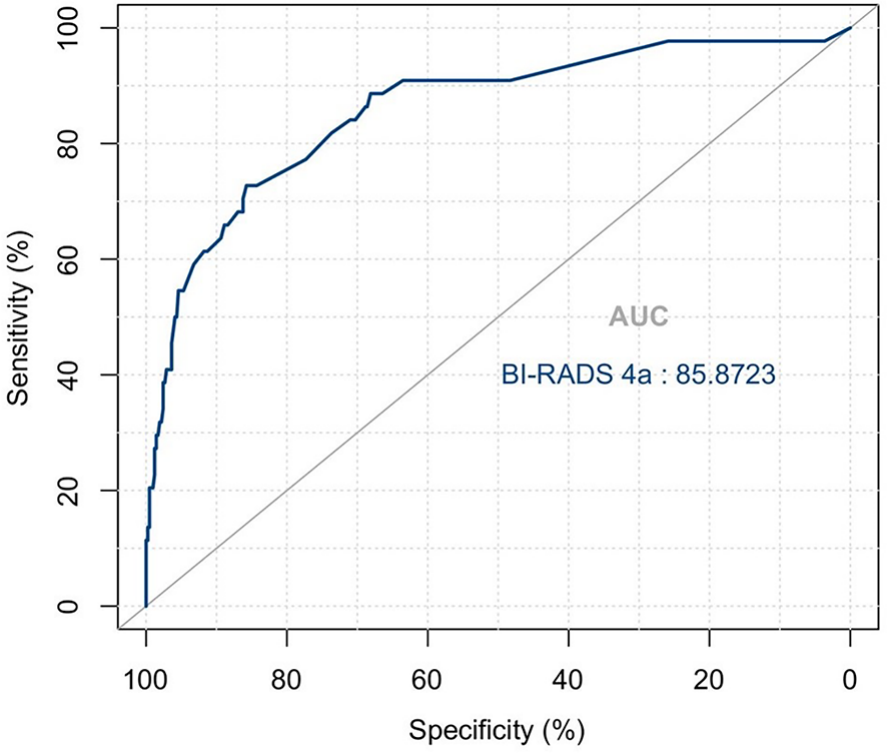
ROC curve for predicting benign and malignant masses in category 4a patients. AUC = 85.9.

**Figure 3:**
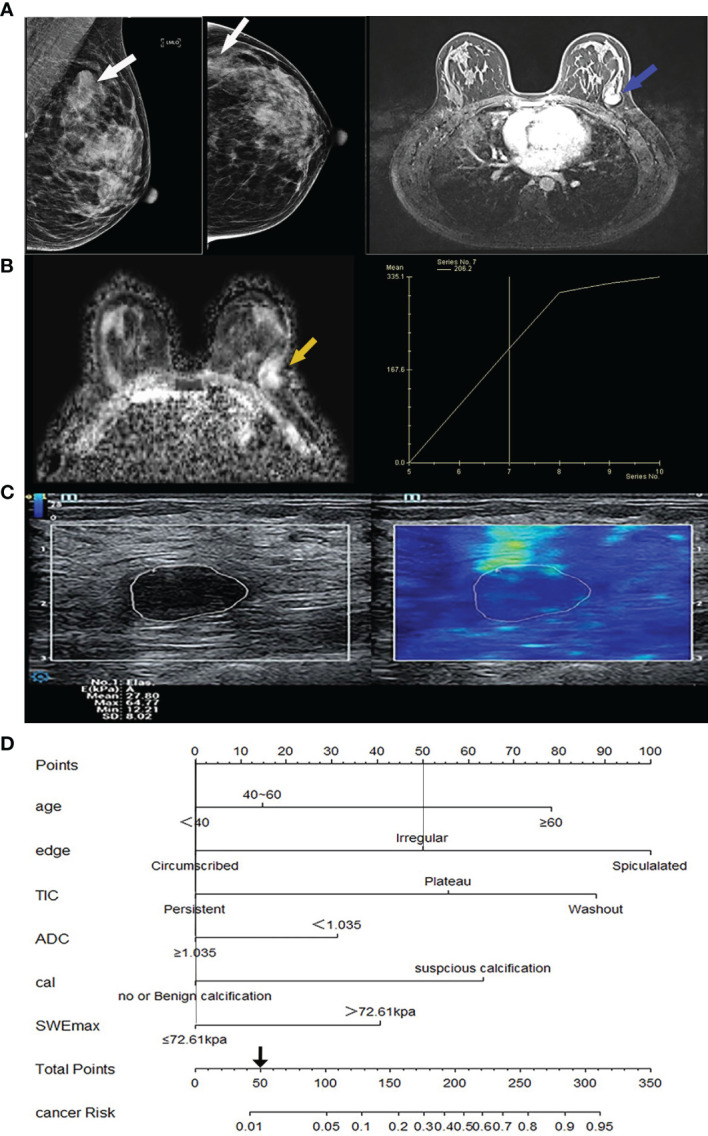
Image from a 32-year-old woman suffering from fibroadenoma BI-RADS category 4A lesions. **(A)** Mammography imaging on the left (lesion indicated by white arrows) and the first phase of magnetic resonance dynamic enhanced transverse axial images on the right (lesion indicated by blue arrows) showed a lobulated mass near the chest wall, without calcification. **(B)** Magnetic resonance transverse axial ADC image showed a high signal value of 1.4 × 10^-3^ mm^2^/s of the lesion on the left (indicated by yellow arrows) with ascending TIC curve on the right. **(C)** The ultrasound shear wave elastography shows SWEmax = 64.77 KPa. **(D)** In the comprehensive score of Nomogram, only the marginal irregularity (lobed) accounted for 50 points, which was less than 106 points, and it was downgraded into BI-RADS 3 category. cal, calcification morphology; TIC, time-signal intensity curve; ADC, apparent diffusion coefficient.

The variables were assessed in a univariable logistic regression analysis, and the variables with outcomes of *p* < 0.05 were entered into multivariable logistic regression. The results in **Table 3** showed that TIC curve (*p* = 0.000), ADC value (*p* = 0.043), mass margin (*p* = 0.018), calcification morphology (*p* = 0.000), SWE max (*p* = 0.024), and age groups (*p* = 0.000) were independent variables for differentiating between benign and malignant tumors, and DWI signal is excluded in the multivariable logistic regression analysis, which may have a strong correlation with the ADC value. Display these independent predictors as a nomogram (**Figure 1A**) and the calibration curve (**Figure 1B**) showed floating around the baseline, indicating that the model is suitable well. Then, a straight line is drawn upwards, to the point of the axis on the top, to acquire the points received based on covariates, respectively. Total points are calculated by adding all the points obtained from every covariate. The final sum is located on the total points axis, and a straight line was drawn downwards from there to obtain the probability of risk degree. The ROC curve (**Figure 2**) showed that the AUC of the model was 85.9. Through the nomogram, the cutoff score to distinguish between benign and malignant was 106 points, and the risk degree was 0.063. A patient (**Figure 3**) whose risk is less than 0.063 will be downgraded. Thus, 59.6% (273/458) of patients were downgraded by nomogram, and 4 malignant patients were downgraded to BI-RADS 3 (**Table 4**). The sensitivity of the overall classification of the mass was reduced from 100% to 99.6%, and the specificity was increased from 4.41% to 45.3%. The accuracy increased from 63.4% to 78.8%.

**Table 3:** Differential regression analysis of imaging and clinical indexes of benign and malignant lesions of class 4A (only showing the difference of imaging indexes with statistical significance).

Variables	Univariable logistic analysis		Multivariable logistic analysis	
	ORs (95% CI)	*p*-Value	ORs (95% CI)	*p*-Value
**TIC curve**		0.000*		0.000*
Persistent	1.0 (Reference)		1.0 (Reference)	
Plateau	5.26 (2.42,11.42)	0.000*	4.43 (1.73,11.37	0.002*
Washout	9.88 (4.35,22.45)	0.000*	11.23 (4.13,30.52)	0.000*
**DWI**	2.07 (1.038,4.14)	0.039*	1.77 (0.77,4.07)	0.178
**ADC**	4.56 (2.39,8.71)	0.000*	2.23 (1.02,4.84)	0.043*
**Edge**		0.000*		0.018*
Circumscribed	1.0 (Reference)		1.0 (Reference)	
Irregular	2.70 (0.94,7.80)	0.066	4.46 (1.22,16.32)	0.024*
Spiculated	40.42 (7.05,231.61)	0.000*	18.98 (2.11,170.99)	0.009*
**Calcifications**	3.53 (1.72,7.24)	0.001*	5.76 (2.30,14.43)	0.000*
**SWEmax**	2.29 (1.14,4.62)	0.02*	2.79 (1.15,6.79)	0.024*
**Age (years)**		0.005*		0.000*
<40	1.0 (Reference)		1.0 (Reference)	
40–60	1.06 (0.48,2.34)	0.889	1.55 (0.62,3.89)	0.349
>60	3.23 (1.37.7.61)	0.007*	9.55 (2.99,30.51)	0.000*
**Tenderness**	1.31 (0.45,3.830	0.620		
**Mass mobility**	1.8 (0.74,4.41)	0.19		
**Family history**	5.26 (0.65,4.84)	0.27		
**Hormone therapy**	0.57 (0.17,1.92)	0.35		
**Breast surgery**	0.78 (0.27,2.28)	0.65		
**Skin changes/Nipple discharge**	0.43 (0.06,3.01)	0.42		

* The significance of the difference between Benign and Malignant. Data in parentheses are 95% confidence intervals. ORs, odds ratios; CI, confidence interval; TIC, time–signal intensity curve; ADC, apparent diffusion coefficient; DWI, diffusion-weighted imaging.

**Table 4:** BI-RADS classification information for eligible patients after downgrade.

		Benign (%)	Malignant (%)	Total
**BI-RADS**	3	298 (98.7)	4 (1.3)	302
	4A	145 (76.7)	40 (21.6)	185
	4B	110 (57.6)	81 (42.4)	191
	4C	91 (16.8)	450 (88.2)	541
	5	14 (2.8)	484 (97.2)	498
	Total	658 (100.0)	1,059 (100.0)	1,717

Unless otherwise indicated, the value is the number of patients and the percentage in parentheses. BI-RADS, Breast Imaging Reporting and Data System.

## Discussion

Breast cancer has become a global disease. The main age of breast cancer in Chinese women is between 45 and 60 years old (7). The increasing detection rate of early breast cancer and suitable treatment has successfully reduced the mortality rate of breast cancer. For patients with no-dense gland breasts, mammography was the preferred examination, but for Asian females with dense gland breasts, ultrasound and magnetic resonance examination proved more advantageous. For this batch of patients who underwent three imaging modalities, the sensitivity of the results was very high, and the specificity was very low.

All patients with a final BI-RADS score of 4 or 5 received a diagnostic core needle biopsy or open surgery was performed to determine the histopathologic diagnosis, as recommended by the American College of Radiology. Previous studies showed how to downgrade BI-RADS 4A relatively safely, to have these patients followed up instead of undergoing an immediate biopsy. For example, Flowers et al. (8) proposed that BI-RADS 4A is defined as a low-risk disease, which can be clinically evaluated and followed up instead of performing a biopsy immediately. The classification interval of benign and malignant masses is between categories 3 and 4A, and because the positive predictive value (PPV) of BI-RADS 4A patients is less than 10%, a large part of the pathology after biopsy is confirmed to be benign, leading to an unnecessary invasive examination. Therefore, the author believes that it is necessary to further analyze which indicators are different between benign and malignant masses, which patients are suitable for short-term follow-up, and which are suitable for biopsy, to establish a predictive model of risk factors in category 4A patients. This study is based on Chinese samples; the results showed that 273/458 (59.6%) of BI-RADS 4A patients could be degraded and the malignant rate of the degraded patients was 4/273 (1.5%); the histopathological types of 4 false-negative patients were small B-cell lymphoma, ductal epithelial dysplasia, low-grade ductal carcinoma *in situ*, and solid papillary carcinoma *in situ*. Small B-cell lymphomas in hematological diseases can be differentiated based on medical history, and the remaining 3 missed diagnoses can be treated according to the progress of the disease during regular follow-up. Especially for young patients less than 40 years old, 73.7% (101/137) were downgraded to BI-RADS 3. Only one case was wrongly degraded, and the pathological type was *in situ* solid papillary carcinoma. The overall accuracy, especially the specificity, can be significantly improved without significantly reducing the sensitivity.

Selecting the malignant signs with the highest risk to construct a nomogram can help distinguish benign and malignant lesions and improve the diagnostic value. Among them, the TIC curve, ADC value, mass edge, calcification morphology, age, and SWEmax were identified as independent predictors of benign and malignant tumors, which are the same as the previous study (9–11). No matter from the multivariable logistic regression analysis or the visualization of the nomogram, it can be seen that the edge spiculated of the tumor is the most powerful indicator to predict the malignant tumor. The malignant risk of masses with spiculated edge is 18.98 times that of clear margin. Because the malignant mass grows to infiltrate, the formation of traction on the surrounding tissue can be manifested as a spiculated sign. In addition to morphological characteristics, hemodynamic characteristics also play an important role in predicting benign and malignant masses. Jiang (10) believes that the TIC curve can objectively and accurately assess the dynamic enhancement characteristics of the diseased tissue, and has high specificity and sensitivity for the differential diagnosis of breast diseases. The risk of malignancy of the washout TIC curve is 11.23 times that of the non-enhanced or continuously rising TIC curve. Similarly, ADC values are used to visualize and quantify the random movement of water molecules in human tissues. Studies have shown that the ADC value can distinguish malignant and benign breast lesions and improve diagnostic specificity (11). Breast cancer is usually expressed as a low ADC image signal, which is attributed to the increase of cell density, and the restriction of the diffusion of water molecules due to changes in the microstructure of the cells. SWEmax is related to benign and malignant tumors (12). In the new version of the BI-RADS guidelines, elastography has become a useful tool for breast examination and tumor assessment. Meta-analysis shows that elastography can help differentiate benign from malignant breast lesions, improve the diagnostic accuracy of malignant breast lesions, and reduce unnecessary breast biopsy (13).

In our study, among the clinical factors, the patient’s age is the most significant predictor, while other factors (such as tenderness, mass activity, hormone therapy, history of breast surgery, skin changes/nipple discharge, and family history of breast cancer) have no significant difference between benign and malignant tumors. This may be due to the large proportion of benign lesions in patients with category 4A, the unobvious clinical manifestations, or the limited sample size. Research by Jagpreet (14) also confirmed that family history of breast cancer or hormone use was not an important predictor of breast cancer, and the risk of breast cancer increased with age. Among the selected 4A patients, malignant risk in patients from the 40 to 60 years old group and older than 60 years old group is 1.55 times and 9.55 times that of the less than 40 years old group. According to the BI-RADS 4 category, it can be divided into 4A, 4B, and 4C subtypes (15, 16). It is found that there is a positive correlation between the malignancy rate of each subtype and the age group, and the difference is statistically significant. Similarly, Raza (17) also found that age is an important clinical factor in predicting malignant tumors. They suggested that for older patients, the threshold of biopsy should be lowered, and even biopsy should be performed on tumors with benign imaging features. It was also reported that the malignant rate of category 3 of nodules was more than 2% in patients over 60 years old (6), which further proved that age was the most important clinical factor influencing the benign and malignant masses in any category of mass. Our research divides the age of BI-RADS 4A patients into 3 groups (malignant rate): <40 years old (7.3%); 40–60 years old (7.7%); >60 years old (20.7%) (*p* < 0.001), which proves that older is an important risk factor for breast cancer. Our results were consistent with the above previous studies. There were currently a variety of breast cancer risk assessment models, and the existing risk prediction models were generally similar (18–20).

This study has some limitations. First of all, this article only degrades the patients who are classified as BI-RADS category 4A. Because even if patients above category 4A are degraded, they are still within the scope of biopsy. Therefore, patients of categories 4B, 4C, and 5 are not considered in our study. Secondly, we did not evaluate the consistency between observers, but previous studies have shown that the feasibility of guidelines makes the results of the report not significantly different between junior and experienced radiologists (15). Finally, this study did not include the immunohistochemical results to predict the model because these factors are obtained after biopsy, which may limit the clinical application of the model.

## Conclusion

In short, by combining the BI-RADS lexicon and clinical indicators to perform downgrading for BI-RADS 4A patients, a large number of patients can be prevented from undergoing invasive biopsy, and clinical resources can be saved.

## Data Availability Statement

The raw data supporting the conclusions of this article will be made available by the authors, without undue reservation.

## Ethics Statement

The studies involving human participants were reviewed and approved by the Ethics Committee of the Ruijin Hospital, Shanghai Jiao Tong University School of Medicine. Written informed consent for participation was not required for this study in accordance with the national legislation and the institutional requirements.

## Author Contributions

XZ, WZ, and WC contributed to conception and design of the study. YX performed the statistical analysis. YX wrote the first draft of the manuscript. SZ, SX, YZ, and XZ wrote sections of the manuscript. All authors contributed to manuscript revision, read, and approved the submitted version.

## Conflict of Interest

The authors declare that the research was conducted in the absence of any commercial or financial relationships that could be construed as a potential conflict of interest.

## Publisher’s Note

All claims expressed in this article are solely those of the authors and do not necessarily represent those of their affiliated organizations, or those of the publisher, the editors and the reviewers. Any product that may be evaluated in this article, or claim that may be made by its manufacturer, is not guaranteed or endorsed by the publisher.
